# Comparison of genomic prediction accuracies in dairy cattle lactation traits using five classes of functional variants versus generic SNP

**DOI:** 10.1186/s12711-025-00966-2

**Published:** 2025-04-11

**Authors:** Setegn Worku Alemu, Thomas J. Lopdell, Alexander J. Trevarton, Russell G. Snell, Mathew D. Littlejohn, Dorian J. Garrick

**Affiliations:** 1 AL Rae Centre for Genetics and Breeding, Massey University, 10 Bisley Drive, Hamilton, 3240 New Zealand; 2 Invermay Agricultural Centre, AgResearch Limited, Mosgiel, New Zealand; 3 LIC, Hamilton, New Zealand; 4 School of Biological Sciences, University of Auckland, Auckland, New Zealand

## Abstract

**Background:**

Genomic selection, typically employing genetic markers from SNP chips, is routine in modern dairy cattle breeding. This study assessed the impact of functional sequence variants on genomic prediction accuracy relative to 50 k SNP chip markers for fat percent, protein percent, milk volume, fat yield, and protein yield in lactating dairy cattle. The functional variants were identified through GWAS, RNA-seq, Histone modification ChIP-seq, ATAC-seq, or were coding variants. The genomic prediction accuracy obtained using each class of functional variants was compared with matched numbers of SNPs randomly selected from the Illumina 50 k SNP chip.

**Results:**

The investigation revealed that variants identified by GWAS or RNA-seq, significantly improved the prediction accuracy across all five traits. Contributions from ChIP-seq, ATAC-seq, and coding variants varied. Some variants identified using ChIP-seq showed marked improvements, while others reduced accuracy in protein yield predictions. Relative to a matched number of 32,595 SNPs from the SNP chip, pooling all the functional variants demonstrated prediction accuracy increases of 1.76% for fat percent, 2.97% for protein percent, 0.51% for milk volume, and 0.26% for fat yield, but with a slight decrease of 0.43% in protein yield.

**Conclusion:**

The study demonstrates that functional variants can improve prediction accuracy relative to equivalent numbers of variants from a generic SNP panel, with percent traits showing more significant gains than yield traits. The main advantage of using functional variants for genomic prediction was achievement of comparable accuracy using a smaller, more selective set of loci. This is particularly evident in trait-specific scenarios. Our findings indicate that specific combinations of functional variants comprising 16 k variants can achieve genomic prediction accuracy comparable to employing a standard panel of twice the size (32.6 k), especially for percent traits. This highlights the potential for the development of more efficient, trait-focused SNP panels utilizing functional variants.

**Supplementary Information:**

The online version contains supplementary material available at 10.1186/s12711-025-00966-2.

## Background

Genomic selection, an approach now extensively employed in both animal and plant breeding programs, depends on the use of markers. This technique can be applied to complex traits, in which case it usually employs a large number of markers dispersed throughout the genome to estimate an individual’s genetic value, or Genomic Estimated Breeding Value (GEBV) [1]. The accuracy of GEBVs, particularly for young animals without individual phenotypes, surpasses the accuracy of EBVs obtained using only traditional pedigree methods [2]. This may allow a reduction in generation interval in some circumstances, especially in comparison to progeny-test based selection in dairy cattle, potentially doubling the rate of genetic gain [3, 4].

Emerging research suggests that the accuracy of genomic prediction can be enhanced through the incorporation of causal variants [5, 6]. However, previous studies using whole-genome sequence-derived causal variants demonstrated only limited improvement to the accuracy of genomic prediction [7, 8]. This limited improvement could stem from the large standard errors in marker effect estimation, particularly when the training population is small [7, 8]. Furthermore, when all sequence data are fitted simultaneously, long-range linkage disequilibrium could mask the true location of the underlying Quantitative Trait Nucleotide (QTN) [7, 8]. This can lead to erroneous assignment of causal effects to non-causal variants in LD with the true QTN. Such misallocation of effects can introduce bias and reduce the model’s predictive power, particularly when applied to different populations where LD patterns may vary. The challenge associated with using whole-genome sequence data is compounded relative to SNP chip markers due to the substantial costs and computational demands.

A potentially viable alternative is to select an informative set of variants, such as variants with predicted functional effects to attempt to enhance genomic prediction accuracy. Functional variants that have been imputed into high-density SNP genotypes have demonstrated an improved genomic prediction accuracy in sheep and dairy cattle [6, 9, 10]. However, despite some promising results, the application of functional variants in genomic prediction is still in its developmental stages. Genome-wide association studies (GWAS) targeting traits of interest are often conducted to identify functional variants. These studies facilitate the identification of variants that are relevant to the traits being investigated [6, 9, 10]. These variants could enhance genomic prediction for new populations when genetic information from diverse breeds is incorporated. It is important to note that this strategy is distinct from selection based only on annotation. Here, the chosen variants have established empirical associations with the traits of interest as well as being supported by evidence from annotation, thereby increasing their relevance to the traits under consideration.

The selection of functional variants can be extended to include those that impact molecular rather than production phenotypes, as QTL for molecular traits are hypothesized to underlie the genetic signals observed in more complex traits [11]. Molecular QTL can be identified from a variety of sequencing technologies, though perhaps most commonly they come from the implementation of RNA sequencing techniques (RNA-seq) to construct molecular phenotypes. RNA-seq data provides an extensive profile of the transcriptome in a particular tissue at a particular time, including gene expression levels, alternative splicing events, and post-transcriptional modifications. By capturing the underlying regulatory mechanisms, RNA-seq data might help identify variants that will improve genomic predictions. Moreover, RNA-seq data may highlight expression quantitative trait loci (eQTLs), allowing the identification of tag variants of modulator loci that may impact complex phenotypes [12]. This approach has shown significant potential for enhancing genomic prediction accuracy in dairy cattle [13]. In further support of this approach, it was observed that SNPs within eQTL or splicing QTL account for over two-thirds of heritability, on average, across various traits [14].

Chromatin Immunoprecipitation Sequencing (ChIP-seq) data representing histone modifications serves as another potential source of molecular QTL tag variants. Histone modifications play a pivotal role in regulating gene expression by altering the accessibility of functional elements in the DNA to transcription factors and other regulatory factors. By identifying variants associated with histone modifications, QTL can be identified that influence gene activity and, consequently, complex traits in dairy cattle [15]. Chromatin accessibility can be assayed more directly using the Assay for Transposase-Accessible Chromatin with Sequencing (ATAC-seq), and variants within ATAC-seq peaks might be expected to be useful for genomic selection. In addition to the regulatory and expression variants identified by histone modification ChIP-seq and ATAC-seq, incorporating coding variants, which are identified through sequencing, could further enhance genomic prediction accuracy. By including variants that alter the amino acid sequence of proteins, inclusion of these variants may capture protein function effects distinct to the regulatory impacts assayed by RNA-seq, ChIP-seq, and ATAC-seq.

This paper investigates the influence of putative functional variants identified using five different methodologies: GWAS, RNA-seq, histone modification ChIP-seq, ATAC-seq variants, and coding variants. Additionally, we have tested combinations of variant categories to ascertain the collective efficacy of these functional variant sets.

Specifically, our primary aim was to assess the impact of tag and putative functional variants on the accuracy of genomic prediction, when benchmarked to equivalent numbers of ‘random’ variants from a public bovine Illumina 50 k SNP chip. We assess five important traits in lactating dairy cattle: fat percent, protein percent, milk volume, fat yield, and protein yield, with functional variants covering a range of genomic annotations and categories, including both coding and non-coding variants assumed to have regulatory effects on genes.

## Methods

### Study population, animals, and milk samples

First lactation phenotypes based on commercially collected herd test records were adjusted for non-genetic effects to construct yield deviations (YD) for 494,963 cows included in animal evaluation at Livestock Improvement Corporation (LIC). Routine herd testing typically involves alternate monthly herd testing in which representative subsamples of milk are collected and weighed to determine milk volumes at AM and PM milkings, then pooled and subjected to Fourier-Transform Mid-Infrared (FT-MIR) spectroscopy to estimate 24-h fat percent and protein percent. Fat and protein yield were estimated by multiplying the respective estimates of percent traits by the estimated 24-h milk volume.

### Genotype calling

Genotypes for functional variants were extracted from imputed sequence variants for (n = 166,664) cows, using the method and imputation reference sets previously described in [16]. In brief, genotypes from a variety of lower density SNP chip panels were successively imputed to Illumina 777 k density based on a reference population of 3769 animals genotyped on the 777 k chip, then imputed in a subsequent step to whole genome sequence (WGS) density using a reference of 1298 animals with individual whole genome sequence data at an average depth of 15 ×. After filtering to remove variants with dosage R^2^ (as calculated by Beagle) less than 0.9, this yielded 16.1 million variants with a mean dosage R^2^ of 0.995. Subsets of functional variants were identified within this dataset based on the criteria described in the following sections. Some 91,214 of the 166,664 genotyped cows with yield deviation phenotypes, that had not been used in the discovery of any of the functional variants, were used to compare predictive performance in this study. These animals comprised 21,584 Holstein-Friesians, as defined by having ≥ 15/16ths of their ancestry recorded to this breed based on pedigree, as well as 12,292 Jerseys, 45,822 Holstein–Friesian-Jersey cross-bred animals, and 11,516 crosses of other breeds.

### Data analysis

Various systematic genetic factors, such as covariates reflecting ancestry-based coefficients for heterosis, breed, and inbreeding, as well as non-genetic factors such as contemporary group, breed × age, test day mean and Legendre polynomials describing the effects of days in milk were fitted as fixed effects and used to adjust the raw phenotypes to produce yield deviations for subsequent analysis. The adjustment of cow phenotypes involved the following equation:

1 $$\mathbf{y}=\mathbf{X}{\varvec{\upbeta}}+\mathbf{e}$$

In this equation, $\mathbf{y}$ represents a vector of phenotypes, and ${\varvec{\upbeta}}$ represents the fixed class and covariate effects listed above. By employing this equation, the residuals or adjusted phenotypes ($\mathbf{y}-\mathbf{X}\widehat{{\varvec{\upbeta}}}$) were obtained and subsequently utilized for the genomic evaluation.

Inference was based on Markov chain Monte Carlo (MCMC) samples, with each chain consisting of 300,000 samples. Subsamples were saved every 200 iterations, following a burn-in period where the initial 50,000 samples in each sequence were discarded. The genomic evaluation was conducted by fitting a BayesCpi model [17] implemented using Gibb’s sampling in JWAS [18]. To assess the performance of each class of functional variants, a five-fold cross-validation approach was employed. This involved five mutually exclusive validation datasets, where each comprised approximately 20% of the 91,215 genotyped cows (n = 18,243) constructed by random sampling without replacement. For each of the five validation datasets, the remaining 80% of the data comprising the other four datasets (n = 72,972) served as the training population, which was used to estimate the marker effects that were used to predict breeding values for the animals in the validation dataset.

The average correlation between the estimated genomic breeding values and the adjusted phenotypes in the test population was computed to quantify the genomic prediction accuracy. This accuracy assessment was performed for each of the five traits under investigation.

### GWAS SNP

The data and methodology described in Tiplady et al. [19] were used to conduct GWAS except the reference was based on the newer ARS-UCD1.2 genome rather than the older UMD3.1 genome. The GWAS training population comprised 38,085 animals of mixed breed that were not part of the 91,215 animals used in validation. The phenotypes used were Fourier-transform mid-infrared (FTIR-MIR) predicted milk composition yield deviations for fat percent, protein percent, lactose percent, and solids percent (the sum of fat and protein percent), as well as yields deviations for milk volume, fat, protein, lactose, and solids.

In addition to the milk composition and yield data, spectroscopy data for each of 817 MIR wave numbers were used as traits in the GWAS. Following Tiplady et al. [19], the GWAS was performed iteratively, whereby the top significant variant (p < 1.9 × 10^–10^) on each chromosome (if any) was fitted as an additional fixed effect in the next iteration, until no new significant variants remained. Across all phenotypes and iterations, this approach yielded 28,442 significant hits, representing 1847 distinct tag variants.

### RNA-seq data and QTL mapping

Mammary biopsies were collected from 411 F2 Friesian-Jersey cows [20, 21], and RNA sequencing was conducted as previously described. The RNA-seq involved high-depth sequencing on an Illumina HiSeq 2000 instrument, generating 100 bp paired-end reads with two samples multiplexed per lane.

**Fig. 1:**
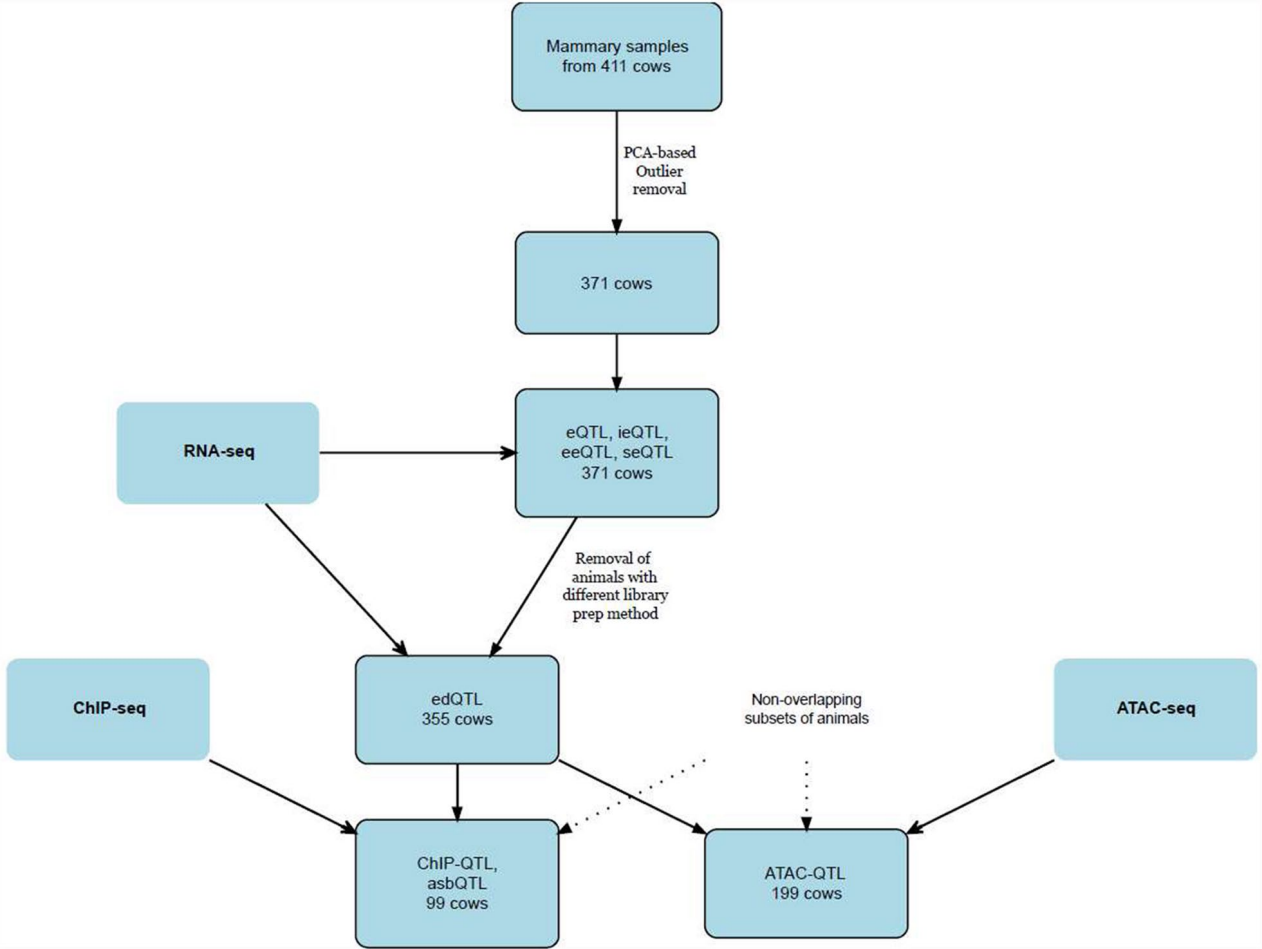
An overview of the animal sets and sequencing technologies used to derive each QTL dataset

Figure 1 provides an overview of the sample processing and analysis workflow. Starting with mammary biopsies from 411 F2 Friesian-Jersey cows, PCA-based quality control resulted in 371 animals being retained for primary QTL analyses. These samples were used for various QTL mappings including expression QTL (eQTL), intronic expression QTL (ieQTL), exon expression QTL (eeQTL), and splicing efficiency QTL (seQTL). A subset of 355 cows, after removing samples with different library preparation methods, was used for RNA editing QTL (edQTL) analysis. The workflow further illustrates the non-overlapping subsets of animals used for different sequencing approaches: 99 cows were selected for ChIP-seq analysis leading to ChIP-QTL and allele-specific binding QTL (asbQTL), while a separate group of 199 cows was used for ATAC-seq analysis resulting in ATAC-QTL identification.

Before mapping, the reads underwent pre-processing using Trimmomatic (version 0.39; [22]) in paired-end mode. To facilitate downstream allele-specific analyses, the processed reads were then mapped to a masked ARS-UCD1.2 genome (where known variants were replaced by bases differing from both alleles), using STAR (version 2.7.0; [23]), in two stages. Initially, the mapping utilized exon and junction information from the RefSeq database (annotation release 106). That mapping was further used to identify novel exons and splice junctions for remapping in a second stage. On average, each cow had approximately 39 million uniquely mapped read pairs.

For gene expression analyses, reads mapped to each gene in the RefSeq database were counted using the featureCounts function of the Subread software package [24]. Genes with a median read count of less than five across samples were excluded. The remaining expression data were processed using the Bioconductor package DESeq2 [25], and principal component analysis (PCA) was performed to detect and exclude outlier samples, resulting in 371 being retained for further analysis. An expression-QTL (eQTL) analysis was conducted using the Mixed Linear-model Association Leaving One Chromosome Out (MLMA-LOCO) approach implemented in GCTA, with imputed sequence genotypes within 1 Mb of the gene used for identifying cis eQTL effects. Taking the most significant variant from each significant QTL (p < 1 × 10^–6^) as a tag, and considering only variants with MAF > 0.1, yielded 4042 distinct variants.

Individual exon-based read counts were recounted for the same 371 animals referenced in the previous paragraph to facilitate the discovery of exon-eQTL (eeQTL). Exons with a median read count of less than five were excluded. Exon expression phenotypes were generated by counting reads mapping to each individual exon using featureCounts, then transformed and adjusted for sequencing batch effects, as described for the eQTL analysis above. The eeQTL analysis was conducted using GCTA-LOCO as per the eQTL analysis described above. Tag variants were identified with p < 1 × 10^–8^ and MAF > 0.1, yielding 7229 distinct variants across all exons.

Impacts of variants on mRNA splicing were explored by identifying QTL for pre-mRNA level expression (intronic expression QTL, or ieQTL) and splicing efficiency (seQTL), analogous to the experiment reported in [26] but extended to the whole genome. For each gene, mapped reads that crossed an intron–exon boundary (using the same gene annotation as used for the eQTL analysis described above) were classified as either spliced or unspliced. The phenotype for the ieQTL analysis was derived by counting the unspliced reads, then proceeding with the VST as per the eQTL analysis. For splicing efficiency, the phenotype was derived as the proportion of reads that spliced out of all reads crossing a splice junction, transformed by the logit function to approximate a normal distribution more closely. The seQTL analysis was then conducted using this phenotype with the same methodology as the eQTL and ieQTL analyses. Applying a significance threshold of p < 1 × 10^–6^, the ieQTL and seQTL yielded 1856 and 821 distinct variants across genes, respectively.

The final dataset created using the RNA-seq data was derived by identifying mRNA editing sites, as reported in [27]. Briefly, nine RNA-seq cows were sequenced with WGS, and the resulting data set used to identify 2413 mRNA editing sites, of which 152 sites showed significant editing QTL (edQTL). From these, 115 unique tag variants were identified and remapped for this study from the UMD3.1 reference genome to ARS-UCD1.2.

### Histone modification ChIP-seq data and QTL

Functional variants selected to represent active histone markers (H3K4Me1, H3K4Me3, and H3K27ac) were the same as those highlighted as ChIP-seq QTL tag variants previously [28]. These markers are associated with active gene regulation: H3K4Me1 marks enhancers, H3K4Me3 marks active promoters, and H3K27ac marks active enhancers and promoters. Briefly, this dataset was derived from 99 duplicate biopsies collected on the same animals sampled for gene expression analyses [20, 21], targeting the histone markers H3K4Me1, H3K4Me3, and H3K27ac [28]. Chromatin was prepared using the Magnify Chromatin Immunoprecipitation kit (ThermoFisher) and sheared to 200–500 bp using the Covaris S2 instrument. Chromatin immunoprecipitation was carried out using the Magnify Chromatin Immunoprecipitation kit with modifications. The immunoprecipitated samples were combined after de-crosslinking, and sequencing libraries were prepared using the NEBNext Ultra II DNA Library Prep Kit for Illumina. The libraries were sequenced on the HiSeq 3000 platform.

Each library was sequenced to yield between 20 and 200 million reads, with a median of 58 million reads. Raw sequence reads underwent adapter and low-quality end trimming using Trimmomatic. Trimmed reads were mapped to a masked ARS-UCD1.2 genome using BWA-MEM (version 0.7.17-r1188; [29]) and poor-quality reads were discarded. MACS2 (version 2.1.1; [30]) was employed to identify peaks from the mapped ChIP-seq reads. Phenotypes for ChIP-seq QTL discovery were created for each ChIP-seq peak, based on the number of reads mapping into each peak, and also the number of reads from, low-depth WGS (≈10 ×) on the input. Phenotype creation proceeded as described in [28]. Associations were identified using the MLMA-LOCO approach implemented in GCTA, incorporating a covariate for sequencing batch. This yielded 335, 3552, and 2234 unique tag variants, respectively, for the H3K27ac, H3K4Me1, and H3K4Me3 datasets.

### Allele-specific expression and binding QTL

Identification of QTL for allele-specific expression (aseQTL), related to exon read counts, was undertaken as per [28]. The same methodology was employed using the ChIP-seq peak read counts to identify allele-specific binding QTL (asbQTL). Briefly, SNPs that were associated with paternal or maternal allele counts were identified using a series of two tests, The first employing a Z-statistic, then those variants with a p-value < 0.001 were fitted in a linear model against the log-ratio of maternal and paternal allele counts. After filtering for p < 1 × 10^–8^ and MAF > 0.1, this yielded 5625 distinct aseQTL variants. Applying a less stringent threshold of p < 1 × 10^–6^ to the asbQTL yielded 252 H3K27ac peak variants, 3065 for H3K4Me1, and 3620 for H3K4Me3.

### ATAC-QTL

A subset of 199 RNA-seq animals, not overlapping with the 99 animals analysed using ChIP-seq, was used in an Assay for Transposase-Accessible Chromatin with Sequencing (ATAC-seq) experiment, as described in [31]. Briefly, ATAC-seq libraries were prepared using the Active Motif ATAC-Seq kit (Active Motif, Carlsbad, California, USA) then sequenced on an Illumina Novaseq machine. Reads were mapped to the same masked reference genome as used in the ChIP-seq analysis, and peaks called using MACS3 using a consensus alignment comprising a sample of 5% of the reads from each individual animal. Phenotyping and QTL discovery proceeded analogously to the ChIP-seq experiment, as described in [31]. Across all significant peaks (p < 1 × 10–8) in the genome, this yielded a total of 12,303 unique tag variants.

### Coding variants

Coding variants were identified from sequence variants using Ensembl’s Variant Effect Predictor (VEP release 97, [32]). A total of 195,760 variants with predicted moderate or high effects were identified in the WGS reference population. These were filtered further to keep only variants with MAF > 0.025, average sequence depth > 10 per animal, and read mapping quality > 40 (out of a maximum of 60 for BWA-MEM). The genes to which the variants mapped were subsequently filtered by looking up the ratio of observed to expected SNVs in gnomAD (v2.1.1, [33]) for missense or pLoF according to whether the VEP prediction was MODERATE or HIGH effect respectively. To focus on the variants most likely to be deleterious, variants located in genes with o/e ratio > 0.9 were removed. This resulted in a final set of 2515 predicted deleterious coding variants, comprising 1937 missense variants, 274 frameshift variants, 162 stop gains, 57 splice donors, 37 splice acceptors, 30 start lost, and 18 stop lost.

### Final variant set selection

Version one of the Illumina BovineSNP50 50 K SNP panel consisted of 54,001 SNPs which was originally constructed to avoid exomic or functional variants. From these, a set of 34,927 autosomal variants originally selected for commercial genomic prediction were tested as the benchmark set. These variants had been previously filtered to remove markers with MAF < 0.02, low call rates (< 0.9), high linkage disequilibrium (R^2^ > 0.9 removed), and deviation from Hardy–Weinberg equilibrium (those with a p < 0.15 were removed). To enable a balanced comparison with our functional variant set, we randomly selected 32,595 SNPs from this filtered set of 34,927 SNPs, hereafter referred to as the ‘current SNP panel’. This panel served as our reference standard for comparison with all functional variant groups.

A total of 43,319 distinct tag or causal variants were identified across all the sequence-based functional variant sets described above. To reduce the variant set to match the number and MAF spectrum of the quality-filtered SNPs from the 50 k panel (i.e. ‘current SNP panel’; N = 34,927 variants), we first allocated each variant to linkage-disequilibrium (LD) blocks with a threshold of R2 = 0.98, then selected one variant to represent each block, prioritising those from gene expression QTL, coding variants, or GWAS over other annotation classes. This yielded a set of 39,686 variants. The numbers of variants falling within MAF bins of width 0.05 (i.e., [0.00, 0.05), [0.05, 0.10), etc.), were compared to counts determined in the same fashion for the current SNP panel. The functional variant set was then subsampled within each bin, based on the categories and numbers of associated QTL for each variant, to match the current SNP panel bin counts. This resulted in a final set of 34,927 variants with an allele frequency distribution matching that of the current SNP panel variant set. These variants comprised 4463 distinct tags for 45,855 eeQTL (many variants tag eeQTL for multiple exons in the same gene), as well as tags for 1248 GWAS QTL (many variants tag QTL in multiple wavenumbers), 10,368 ATAC-seq QTL, 5900 eeQTL, 5704 asbQTL, 4940 ChIP-seq QTL, 1600 ieQTL, 1499 coding variants, 690 seQTL, and 68 edQTL. Many variants had signals in multiple categories. For further refinement, only biallelic loci were selected and SNPs with minor allele frequencies less than 0.1 were excluded. As a result of this filtering, our set comprised of 1124 GWAS QTL, 9068 ATAC-seq QTL, 5328 eeQTL, 4154 aseQTL, 5682 asbQTL, 4496 ChIP-seq QTL, 903 ieQTL, 1389 coding variants, and 608 seQTL (N = 32,595).

Additionally, we classified the functional variants into combined categories based on their functional characteristics. For example, we grouped RNA-seq-derived variants (eeQTLs, aseQTLs, ieQTLs, and seQTLs) and histone-related variants (ATAC-QTLs and asbQTLs), resulting in 10,576 RNAseq variants and 14,949 histone-related variants. We also created specific combinations of functional variants from GWAS, RNA-seq, and histone-related categories. This led to the identification of three combinations: 6449 SNPs both GWAS and eeQTL, 12,029 SNPs that were GWAS, eeQTL, and asbQTL, and 15,966 SNPs that were GWAS, eeQTL, asbQTL, and aseQTL. Furthermore, all functional variants were combined into a category termed “AllSNP”, totalling 32,595 SNPs.

To evaluate the genomic prediction accuracy of each functional variant set, we compared it with the accuracy obtained by randomly sampling loci across the genome equivalent to the functional variant count from the current SNP panel. For example, within the GWAS functional category, 1157 loci were selected from this current SNP panel. Table 1 displays the functional variants, and their associated SNP counts before and after the filtering process.

**Table 1:** Counts of SNP variants by functional class, initial identified, before and after indel and MAF filtering

Functional class	Variant type	Initially identified	Before indel and MAF	After indels and MAF
GWAS	GWAS	1847	1248	1124
RNA-seq	eeQTL	7229	5900	5328
	aseQTL	5625	4200	4154
	ieQTL	1856	1600	903
	seQTL	821	690	608
Histone-related	asbQTL	6937	5704	5682
	ChIP-seq QTL	6121	4940	4496
	ATAC-QTL	12,303	10,368	9068
	Coding	2515	1499	1389
RNA-seq	eeQTL + aseQTL + ieQTL + seQTL	12,344	11,310	10,576
Histone-related	ATAC-QTL + absQTL + ChIP-seq QTL	25,361	18,523	17,407
	eeQTL + GWAS	9076	7111	6416
	eeQTL + GWAS + asbQTL	16,014	12,712	11,996
	eeQTL + GWAS + asbQTL + aseQTL	21,638	16,658	15,932
	All	43,319	34,927	32,595

The improvement in genomic prediction accuracy with functional variants was determined by contrasting predictive ability with the performance of an equivalent loci count from the Illumina SNP panel. For each functional variant group, an equivalent number of SNPs were randomly sampled from the current SNP panel to serve as a comparison baseline. The improvement in predictive ability was quantified as $100 \times \left(\frac{Functional-Current}{Current}\right)$ where $Functional$ refers to genomic prediction accuracy computed using the given putative functional variant category and $Current$ refers to the corresponding genomic prediction accuracy computed using an equivalent number of randomly selected SNPs from the current SNP panel.

## Results

**Fig. 2:**
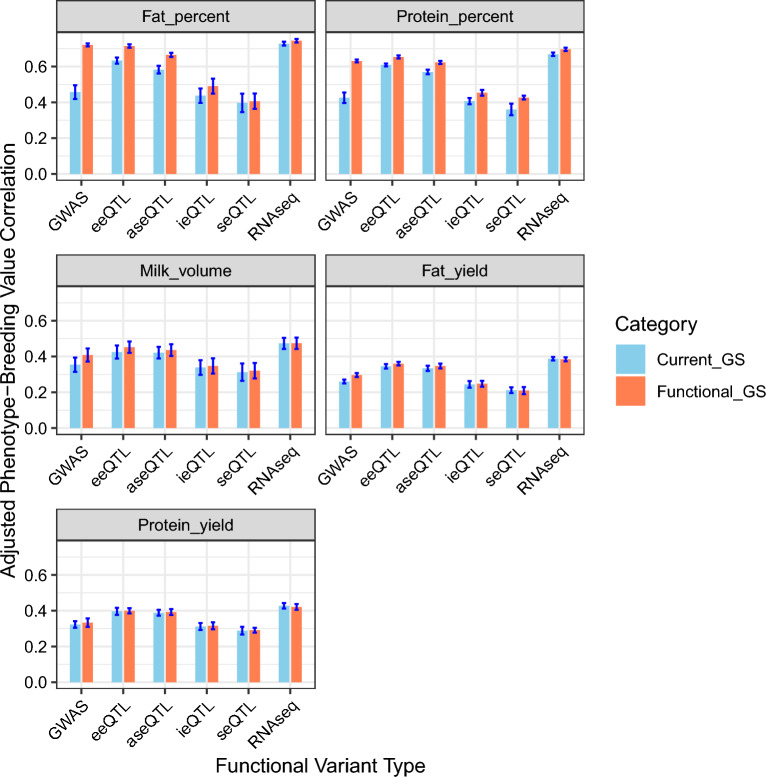
Genomic prediction accuracy using functional variant sets from GWAS and RNA-seq variants (eeQTL, aseQTL, ieQTL and seQTL), plus combined RNA-seq, compared to predictions from similar numbers of loci randomly sampled from the current GS panel

Figure 2 shows the accuracy of genomic prediction for different classes of functional variants. Accuracy here is measured as the correlation between estimated genomic breeding values and the adjusted phenotypes for each trait in the test population. Each prediction is benchmarked against the GEBVs obtained using randomly selected samples of the loci from the current SNP panel.

As shown in Fig. 2, the use of 1124 GWAS-selected functional variants significantly improved the genomic prediction accuracy for all five traits assessed in this study, compared to an equivalent number of randomly selected SNPs from the current SNP panel. Specifically, there was an increase in genomic prediction accuracy by 57.77% for fat percent, 48.28% for protein percent, 15.36% for milk volume, 13.83% for fat yield, and 3.18% for protein yield. These results highlight the advantages of using functional variants over a similar sized set of SNPs from the current SNP panel**.** All results for genomic prediction accuracy for the five traits and all functional variants are presented in Additional file 1 Table S1. Additionally, an interactive web application (https://setegn.shinyapps.io/Functional_genomics/) is available for exploring subsets of SNP types in greater detail. This Shiny app provides comprehensive statistics, including means, standard deviations, and the number of variants for each functional class across all traits. The source code for the Shiny app is publicly available and can be accessed at the following GitHub repository: https://github.com/setegnworku/Functional-genomics.

Similarly, the implementation of RNA-seq functional variants led to significant improvements in genomic prediction accuracy across all five evaluated traits, with the sole exception of fat yield for seQTL tags. Specifically, the eeQTL variants resulted in an accuracy increase of 12.82% for fat percent, 7.26% for protein percent, 6.40% for milk volume, 4.36% for fat yield, and 0.72% for protein yield. Furthermore, the utilization of ieQTL functional variants led to enhancements in accuracy of 12.16% for fat percent, 11.50% for protein percent, 2.65% for milk volume, 1.46% for fat yield, and 1.19% for protein yield. Interestingly, while the use of seQTL variants caused a decrease of 1.11% in the prediction accuracy for fat yield, they demonstrated a significant increase of 18.47% for protein percent, along with an increase in accuracy for fat percent (2.31%), milk volume (2.64%), and protein yield (0.86%), highlighting their potential utility in trait prediction. Meanwhile, aseQTL functional variants resulted in accuracy improvements for all five traits, with an increase of 14.14% for fat percent, 9.42% for protein percent, 3.49% for milk volume, 3.86% for fat yield, and 1.05% for protein yield.

When these four variant sets (eeQTL, aseQTL, seQTL, and ieQTL) were combined into a set of 10,576 RNA-seq-related tag variants, the overall effect varied, leading to increased prediction accuracy for fat percent (2.23%), protein percent (4.14%), and milk volume (0.13%), but a slight decrease for fat yield (0.85%) and for protein yield (1.50%). These results are in comparison to a control group using random loci sampling from current SNP panel, matched in count to the selected RNA-seq loci (n = 10,576).

**Fig. 3:**
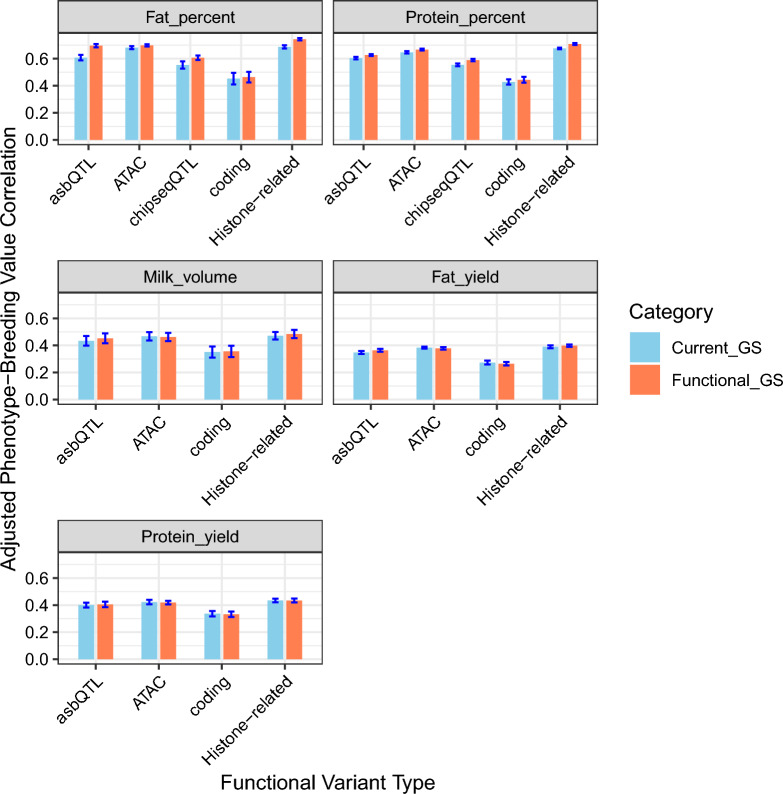
Genomic prediction accuracy using coding variants, plus tag variants for histone modification ChIP-seq, ATAC-seq, and asbQTL (collectively Histone-related variants), compared to similar numbers of loci randomly sampled from the current genomic selection panel

Figure 3 extends the analysis by evaluating the genomic prediction accuracy when incorporating different classes of functional variants, including histone modification ChIP-seq (comprising ChIPseqQTL and asbQTL), ATAC-seq, and coding variants. As shown in Fig. 3, the correlations between estimated genomic breeding values and adjusted phenotypes for each trait in the test population are illustrated, compared with an equal number of SNPs sampled from the current SNP panel.

The use of histone modification ChIP-seq functional variants gave mixed results for genomic prediction accuracy. Notably, the asbQTL class showed improvements in accuracy when compared to an equivalent set of random SNPs from the current SNP panel, demonstrating increases of 14.14% for fat percent, 3.84% for protein percent, 4.29% for milk volume, 4.54% for fat yield, and 1.26% for protein yield. However, the other histone modification ChIP-seq variant class, ChIPseqQTL, showed a slight decrease in genomic prediction accuracy for protein yield (0.56%). Nevertheless, it also exhibited an accuracy improvement of 9.70% for fat percent, 6.40% for protein percent, 2.28% for milk volume, and 0.61% for fat yield. Such findings underscore the potentially significant role of allele-specific binding QTLs in predicting traits.

ATAC-seq functional variants also gave mixed result for genomic prediction accuracy across traits. For percent traits, these variants yielded an improvement in genomic prediction accuracy; however, a slight decrease in accuracy was observed for yield traits when compared against an equivalent number of SNPs sampled from the current SNP panel. Specifically, the enhancements consisted of a 2.44% increase for fat percent and 3.12% for protein percent. On the other hand, a decline of 1.04% was recorded for milk volume, alongside decreases of 0.86% for protein yield and 1.45% for fat yield. Lastly, coding variants yielded minor enhancements in genomic prediction accuracy for some traits, and reductions for others, with improvements of 2.44% for fat percent, 3.79% for protein percent, and 1.31% for milk volume, and reductions of 3.05% in fat yield and 1.26% in protein yield.

When considering the histone-related functional variants collectively (ChIP-seq (comprising ChIPseqQTL and asbQTL) and ATAC-seq), we observed an increase in genomic prediction accuracy, most notably an 8.22% improvement for fat percent, alongside 4.76% for protein percent, 2.73% for milk volume, and 2.07% for fat yield. However, there was a negligible decrease of 0.03% for protein yield.

**Fig. 4:**
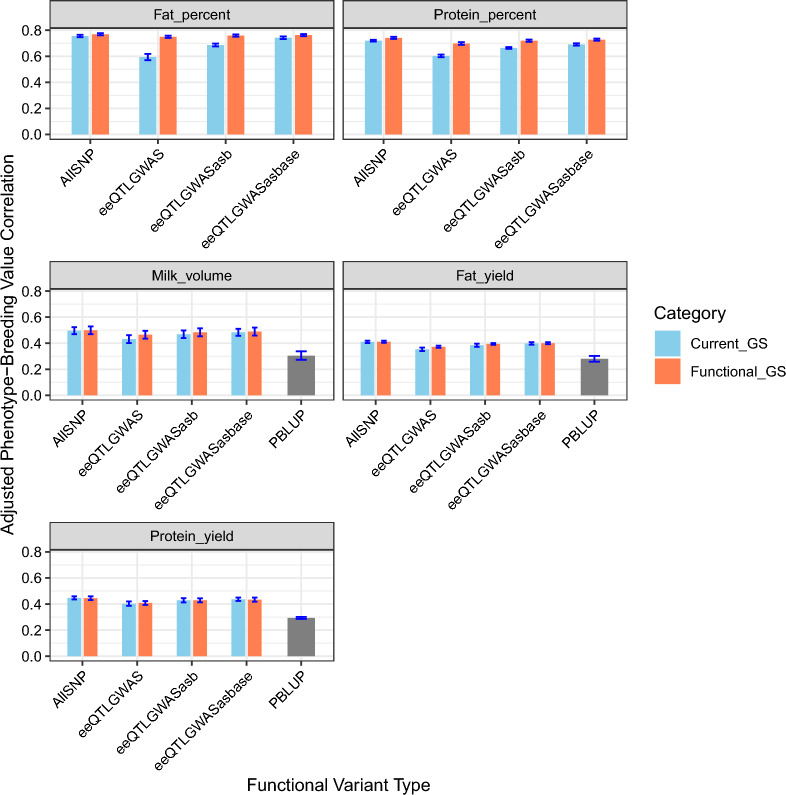
Comparison of genomic prediction accuracy between functional SNP sets and equivalent random samples from current genomic selection panel, including parental average analysis

Expanding upon our initial observations, the analysis depicted in Fig. 4 provides a more detailed examination of genomic prediction accuracy. This is achieved through empirically selected combinations of variants from distinct functional groups, namely GWAS, eeQTL, asbQTL, and aseQTL. This comprehensive approach also considered the aggregated impact of all putative functional variants, collectively labelled as “All”. The specific variant combinations evaluated were “GWAS and eeQTL”, “GWAS, eeQTL, and asbQTL”, and “GWAS, eeQTL, asbQTL, and aseQTL”. Notably, these combinations generally either met or exceeded the genomic prediction accuracy of the full current SNP panel (N = 32,595) for all traits, with the exception of protein yield, while maintaining a SNP count comparable to the current SNP panel.

The combination of “GWAS and eeQTL”, totalling 6416 markers, demonstrated improvements across the five traits. Specifically, there was a 26.17% increase in accuracy for fat percent, 15.70% for protein percent, 7.83% for milk volume, 5.49% for fat yield, and a modest 1.30% for protein yield, compared to an equal number of randomly selected markers from the current SNP panel. Progressing to the combination of “GWAS, eeQTL, and asbQTL”, comprising 11,996 markers an improvement in genomic prediction accuracy was observed. This improvement was quantified as a 10.59% increase for fat percent, 8.41% for protein percent, 3.32% for milk volume, and 2.60% for fat yield. However, it is noteworthy that there was a marginal decrease of 0.11% in protein yield. These metrics were compared with a similar loci count (n = 11,996 markers) from the 32,595 SNP panel. Further, the ensemble of “GWAS, eeQTL, asbQTL, and aseQTL” variants, containing 15,932 markers, yielded an increase in genomic prediction accuracy by 2.70% for fat percent, 5.40% for protein percent, 1.17% for milk volume, and 0.39% for fat yield, alongside a minor decrease of 0.61% in protein yield. This was evaluated against a random sample of loci from the 32,595 SNP panel, equating in count to this ensemble (n = 15,932 markers). Finally, the integration of all functional variants demonstrated an increase in prediction accuracy by 1.76% for fat percent, 2.97% for protein percent, 0.51% for milk volume, and 0.26% for fat yield, but with a slight decrease of 0.43% in protein yield. This was assessed against random loci samples from the 50 K SNP panel, matched in loci count to all functional variant groups (n = 32,595 markers). When compared with the parental average accuracy from PBLUP, which stood at 0.30 for milk volume, 0.28 for fat yield, and 0.29 for protein yield, genomic prediction accuracy was substantially higher in almost all scenarios. For instance, the combination of “GWAS and eeQTL” yielded genomic prediction accuracies of 0.43 for current SNP panel (0.47 for functional GS) for milk volume, 0.35 for current SNP panel (0.37 for functional GS) for fat yield, and 0.40 for current SNP panel (0.41 for functional GS) for protein yield. This clearly demonstrates that family linkage information does not significantly affect the analysis, underscoring the relevance of genomic information in this context.

## Discussion

This primary aim of this study was to investigate the predictive performance of various categories of functionally enriched variants compared to equivalent numbers of variants from a generic commercial SNP panel. Five broad classes of variants were assessed: GWAS, RNA-seq (eeQTL, ieQTL, aseQTL, seQTL), histone modification ChIP-seq (Chip-seqQTL, asbQTL), ATAC-seq, and coding variants. We also evaluated combinations of GWAS, eeQTL, asbQTL, and aseQTL, and explored the effect of combining all variants (“All”). Our results emphasized the influence of these variants on genomic prediction accuracy across five traits in lactating dairy cattle: fat percent, protein percent, milk volume, fat yield, and protein yield.

Among the various functional variants analysed, GWAS tag variants were particularly effective at improving genomic prediction accuracy across all five key traits (fat percent, protein percent, milk volume, fat yield, and protein yield), when compared with equivalent numbers of randomly sampled SNPs from the current SNP panel. The improved accuracy of genomic predictions linked to GWAS variants is assumedly due to their consistent linkage disequilibrium with causal SNPs across different breeds. This observation aligns with previous studies that has shown the advantages of including reliable SNPs from genome-wide association studies to increase genomic prediction accuracy when combined with SNP chip data [9, 34–36]. Furthermore, our findings align with those of other studies [37, 38], emphasizing the importance of carefully choosing SNPs via GWAS to improve genomic prediction accuracy. This finding highlights the substantial role and effectiveness of GWAS-informed SNP selection in increasing genomic prediction accuracy for dairy cattle.

The incorporation of RNA-seq functional variants into genomic prediction models has revealed their capability to refine trait predictions in dairy cattle breeding. Notably, when compared with an equivalent number of randomly sampled SNPs from the current SNP panel, tag variants for eeQTL, ieQTL, and aseQTL exhibited significant improvements in genomic prediction accuracies for the five traits. This consistent improvement across all evaluated traits suggests an important role for these regulatory variants, in controlling various important dairy cattle traits. In contrast, seQTL variants displayed mixed results: they improved predictions for protein percent by 18.47% likely tied to their role in gene splicing processes, as seen in the CSN3 gene [39] but they reduced accuracy in predicting fat yield. Furthermore, when these RNA-seq variants (eeQTL, ieQTL, aseQTL, seQTL) were used in combination, their collective impact was even more pronounced. This aligns with findings in maize research by Guo et al. [40], where integrating various gene expression data significantly increased genomic prediction accuracy, particularly for yield-related traits. In our study, the combined use of RNA-seq variants led to improvements in genomic prediction accuracy in traits such as fat percent, protein percent, and milk volume. However, a decrease in genomic prediction accuracy for fat yield and protein yield was also observed, indicating trait-specific impacts of these variants. Despite these variations, the collective enhancement reaffirms the value of RNA-seq data in identifying complex, trait-associated QTLs, consistent with recent research [14] that highlights the substantial role of gene expression and RNA splicing in the heritability of dairy cattle traits.

The investigation of the effects of histone modification ChIP-seq and ATAC-seq functional variants on genomic prediction accuracy in this study showed variable effects. The asbQTL variant class exhibited an improvement in prediction for all five traits investigated in this study, pointing towards the influence of chromatin conformation on gene regulation in diverse dairy cattle traits. While the histone modification ChIP-seq QTL variant class showed a decrease in accuracy for protein yield prediction, it provided significant improvements in other traits, in fat and protein percent. These observations align with the study conducted by Xiang et al. [6], implies that these traits might be influenced by large-effect variants. On the other hand, the inclusion of ATAC-seq functional variants led to modest yet notable improvements, specifically a 3.12% increase in protein percent, a 2.44% increase in fat percent, and a 0.51% increase in milk volume, demonstrating their potential utility in enhancing genomic prediction accuracy, mainly for the percent traits. However, their performance in predicting fat and protein yield was less remarkable compared to an equivalent number of randomly chosen SNPs from the current SNP panel. Furthermore, combining functional genomic variants related to histones, identified through both histone modification ChIP-seq (including ChIPseqQTL and asbQTL) and ATAC-seq, resulted in improved accuracy for predicting traits like fat percent, protein percent, and milk volume. Using multiple categories of chromatin-related QTL likely captures a broader spectrum of regulatory elements key to influencing gene expression relevant to these traits.

In addition to these findings, coding variants were also found to increase genomic prediction accuracy for milk volume, fat percent, and protein percent. This aligns with the observations of Xiang et al. [6], who suggested an enrichment of large-effect variants within these coding variants for these traits. However, the use of coding variants resulted in a decrease in the prediction accuracy for fat yield and protein yield. This may be due to the absence of large-effect variants for these traits amongst the coding variants. While coding variants can have significant effects, they do not always explain the most heritability [41]. This highlights the importance of considering both the size of the effect and the frequency of variants when assessing their contribution to trait variance.

The combination of functional categories of variants, as shown in Fig. 4, led to some interesting findings. Genomic prediction accuracy improved for all combinations of functional variants across the traits, except for protein yield, when compared to a similar number of SNPs randomly selected from current SNP panel. One interesting point is that the genomic prediction accuracy obtained using the combinations “GWAS, eeQTL, and asbQTL” and “GWAS, eeQTL, asbQTL, and aseQTL” combinations, which consists of 11,996 and 15,932 SNPs respectively, closely matched the accuracy of both the filtered and complete SNP panels. For example, the combination of “GWAS, eeQTL, asbQTL, and aseQTL” slightly outperformed the complete panel for percent traits, generating accuracies of 0.760 for fat percent and 0.730 for protein percent (compared to 0.756 and 0.720 with 34 K SNPs). However, this combination showed slightly lower accuracy for milk volume (0.491 versus 0.496), fat yield (0.40 versus 0.399), and protein yield (0.436 versus 0.447). These results indicate that the genomic prediction accuracy achieved by the current 32,595 SNP chip can be replicated by combining informative functional variants with a lower number of SNPs (< 20 K) from various sources such as GWAS, RNA-seq, and ChIP-seq. Therefore, for these traits, a SNP chip designed using these functional variants (16 K) can generate genomic prediction accuracy comparable to that of the current 33 K SNP panel, particularly for protein percent and fat percent. However, it is important to note that this focused SNP panel may have limited applicability across diverse traits and breeds beyond those studied here. Breeding organizations typically consider a wide range of traits including conformation, health, and fitness, which may not be adequately captured by this panel. Additionally, while a smaller panel might seem cost-effective, developing chips with specific functional variants could potentially increase costs, which may not be economically viable for many breeding organizations. Future research should explore how to balance the precision offered by functional variants with the need for comprehensive trait coverage in breeding programs, possibly by incorporating these variants into existing broad-spectrum SNP panels rather than replacing them entirely. Figure 4 also shows that genomic prediction accuracy increased for four of the five milk traits when all functional variants were combined (32.6 K). However, the enhanced predictive accuracy achieved with the full set of functional variants diminished when compared with specific functional variant categories, such as GWAS and eeQTL. The reason for this might be that genomic prediction accuracy using the current SNP chip might increase when 32,595 SNPs are sampled from a 50 k panel, mainly because a significant portion of the causative SNPs could be in linkage disequilibrium (LD) with the selected SNPs; however, there is still a minor improvement in genomic prediction accuracy in the fat percent and protein percent traits. While our study shows promise in using functional variants for genomic prediction accuracy in dairy cattle for specific traits (protein percent and fat percent), there are practical challenges and limitations associated with this approach. Specifically, sequencing and imputation are essential prerequisites for utilizing functional variants, and errors stemming from these processes can potentially offset the improvements in prediction accuracy. Therefore, before transitioning from current SNP chips to a panel based on imputed functional variants or creating new chips containing these variants, further research is essential to comprehensively assess the trade-offs between potential benefits, logistical complexities, and financial implications.

We observed an improvement in genomic prediction accuracy for protein percent and fat percent across all functional variants, although the degree of improvement varied. This difference in accuracy may be attributed to the fact that percent traits tend to have more precise measurements compared to yield traits. Percent traits are estimated using Fourier Transform Mid-Infrared (FT-MIR) spectroscopy, a measurement technique with inherent but manageable levels of error. Conversely, yield traits are calculated by multiplying the percent traits with milk volume, obtained through herd testing. This measurement of milk volume also carries inherent errors, and when combined with errors from the percent traits, it results in a compounded error (error × error) in the yield trait calculation. This compounded error is reflected in the reduced genomic prediction accuracy of yield trait compared to percent traits and reduces the sensitivity to detect genetic signals. Furthermore, the varying accuracy between percent traits and yield traits could also be influenced by the presence of large-effect QTLs near GWAS SNPs or RNA-seq variants, such as eeQTL and aseQTL. In contrast, traits like milk volume and fat yield showed slight improvements in genomic prediction accuracy, with protein yield even exhibiting a slight decrease. This difference in performance may arise from the existence of multiple QTLs with smaller effects on milk volume and fat yield. These smaller effects are more effectively identified by SNPs dispersed throughout the genome, aligning with findings [5, 6] that highlight the trait-specific nature of advancements in genomic prediction accuracy in dairy cattle. As a result, careful consideration of the traits in question is essential when selecting functional variants for genomic prediction to optimize the benefits derived from these variants. A deeper understanding of how various functional variants impact genomic prediction accuracy allows for the development of more precise and effective breeding strategies tailored to specific traits.

This study demonstrates that genomic prediction utilizing functional variants generally surpasses the performance of the current GS panel. This comparison was conducted using an equivalent number of loci, with SNPs for the panels being randomly chosen from the existing SNP panel. However, further improvement in predictive power using these functional variants could be achieved with advanced statistical models like Neural Network Multilayer Models (NN-MM). These models are adept at capturing complex genetic regulatory sequences, mirroring the data’s layered structure from regulatory and coding sequences to phenotypes [42]. The ability of NN-MM to handle non-linear feature interactions, as indicated in the work of Zhao et al. [43], might offer methodological advantages. Expanding sample sizes in functional variant discovery, especially in histone modification ChIP-seq and ATAC-seq, would reveal additional SNPs, potentially enriching genomic prediction. Investigating other functional variant classes, such as transcribed enhancers and non-coding RNAs, is also promising for increasing prediction accuracy.

## Conclusions

The integration of all functional variants (32,595 SNPs) led to modest improvements in genomic prediction accuracy. Notably, we observed an improvement of 1.76% for fat percent, 2.97% for protein percent, 0.51% for milk volume, and 0.26% for fat yield. However, there was a slight decrease of 0.43% in protein yield compared to using an equivalent number of SNPs from the Illumina 50 k SNP chip. These results, although not substantial, are nonetheless indicative of the value added by using functional variants. Importantly, the research indicates that carefully chosen combinations of functional variants can provide genomic prediction accuracies that match those of more extensive SNP panels, while requiring a significantly smaller number of SNPs (16 k versus 32.6 k). While this finding suggests potential for more focused SNP panels, further research is needed to evaluate their applicability across diverse traits and breeds. Future studies should explore strategies to incorporate these functional variants into existing SNP panels, to potentially increase accuracy for specific traits while maintaining relevance to the diverse, physiologically distinct, traits that are the focus of most breeding programs.

## Supplementary Information

**Additional file 1: Table S1.** Comparison of Genomic Prediction Accuracies in Dairy Cattle Lactation Traits Using Five Classes of Functional Variants Versus Generic SNP. This table compares genomic prediction accuracies across different functional SNP types, including GWAS, eeQTL, seQTL, ieQTL, aseQTL, coding variants, histone-related variants (ChIP-seq, ATAC-seq, asbQTL), and combinations of SNP types (eeQTL + GWAS, eeQTL + GWAS + asbQTL, eeQTL + GWAS + asbQTL + aseQTL, AllSNP) versus generic SNP (randomly sampled SNPs from the current SNP chip, equivalent to functional variant types).

## Data Availability

The datasets used during the current study are privately held by Livestock Improvement Corporation but may be available upon reasonable request.

## References

[1] Meuwissen TH, Hayes BJ, Goddard ME. Prediction of total genetic value using genome-wide dense marker maps. Genetics. 2001;157:1819–29. 11290733 10.1093/genetics/157.4.1819 PMC1461589

[2] Daetwyler HD, Hickey JM, Henshall JM, Dominik S, Gredler B, van der Werf JHJ, et al. Accuracy of estimated genomic breeding values for wool and meat traits in a multi-breed sheep population. Anim Prod Sci. 2010;50:1004–10.

[3] Schaeffer LR. Strategy for applying genome-wide selection in dairy cattle. J Anim Breed Genet. 2006;123:218–23. 16882088 10.1111/j.1439-0388.2006.00595.x

[4] Wiggans GR, Cole JB, Hubbard SM, Sonstegard TS. Genomic selection in dairy cattle: the USDA experience. Annu Rev Anim Biosci. 2017;5:309–27. 27860491 10.1146/annurev-animal-021815-111422

[5] Xiang R, Breen EJ, Prowse-Wilkins CP, Chamberlain AJ, Goddard ME. Bayesian genome-wide analysis of cattle traits using variants with functional and evolutionary significance. Anim Prod Sci. 2021;61:1818–27.

[6] Xiang R, MacLeod IM, Daetwyler HD, de Jong G, O’Connor E, Schrooten C, et al. Genome-wide fine-mapping identifies pleiotropic and functional variants that predict many traits across global cattle populations. Nat Commun. 2021;12:860. 33558518 10.1038/s41467-021-21001-0 PMC7870883

[7] van Binsbergen R, Calus MP, Bink MC, van Eeuwijk FA, Schrooten C, Veerkamp RF. Genomic prediction using imputed whole-genome sequence data in Holstein-Friesian cattle. Genet Sel Evol. 2015;47:71. 26381777 10.1186/s12711-015-0149-x PMC4574568

[8] Veerkamp RF, Bouwman AC, Schrooten C, Calus MP. Genomic prediction using preselected DNA variants from a GWAS with whole-genome sequence data in Holstein-Friesian cattle. Genet Sel Evol. 2016;48:95. 27905878 10.1186/s12711-016-0274-1 PMC5134274

[9] Brondum RF, Su G, Janss L, Sahana G, Guldbrandtsen B, Boichard D, et al. Quantitative trait loci markers derived from whole genome sequence data increases the reliability of genomic prediction. J Dairy Sci. 2015;98:4107–16. 25892697 10.3168/jds.2014-9005

[10] Moghaddar N, Gore KP, Daetwyler HD, Hayes BJ, van der Werf JH. Accuracy of genotype imputation based on random and selected reference sets in purebred and crossbred sheep populations and its effect on accuracy of genomic prediction. Genet Sel Evol. 2015;47:97. 26694131 10.1186/s12711-015-0175-8 PMC4688977

[11] Tiplady KM, Lopdell TJ, Littlejohn MD, Garrick DJ. The evolving role of Fourier-transform mid-infrared spectroscopy in genetic improvement of dairy cattle. J Anim Sci Biotechnol. 2020;11:39. 32322393 10.1186/s40104-020-00445-2 PMC7164258

[12] Kemper KE, Littlejohn MD, Lopdell T, Hayes BJ, Bennett LE, Williams RP, et al. Leveraging genetically simple traits to identify small-effect variants for complex phenotypes. BMC Genomics. 2016;17:858. 27809761 10.1186/s12864-016-3175-3 PMC5094043

[13] Lopdell TJ, Tiplady K, Littlejohn M. Using RNAseq data to improve genomic selection in dairy cattle. In: Proceedings of the World Congress on Genetics Applied to Livestock Production: 11–16 February 2018; Auckland. 2018.

[14] Xiang R, Fang L, Liu S, MacLeod IM, Liu Z, Breen EJ, et al. Gene expression and RNA splicing explain large proportions of the heritability for complex traits in cattle. Cell Genomics. 2023;3: 100385. 37868035 10.1016/j.xgen.2023.100385 PMC10589627

[15] Carey MF, Peterson CL, Smale ST. Chromatin immunoprecipitation (ChIP). Cold Spring Harb Protoc. 2009;2009:pdb.prot5279. 10.1101/pdb.prot5279 20147264

[16] Trebes H, Wang Y, Reynolds E, Tiplady K, Harland C, Lopdell T, et al. Identification of candidate novel production variants on the Bos taurus chromosome X. J Dairy Sci. 2023;106:7799–815. 37562645 10.3168/jds.2022-23095

[17] Habier D, Fernando RL, Kizilkaya K, Garrick DJ. Extension of the Bayesian alphabet for genomic selection. BMC Bioinformatics. 2011;12:186. 21605355 10.1186/1471-2105-12-186 PMC3144464

[18] Cheng H, Fernando R, Garrick D. JWAS: Julia implementation of whole-genome analysis software. In: Proceedings of the World Congress on Genetics Applied to Livestock Production: 11–16 February 2018; Auckland. 2018.

[19] Tiplady KM, Lopdell TJ, Reynolds E, Sherlock RG, Keehan M, Johnson TJ, et al. Sequence-based genome-wide association study of individual milk mid-infrared wavenumbers in mixed-breed dairy cattle. Genet Sel Evol. 2021;53:62. 34284721 10.1186/s12711-021-00648-9 PMC8290608

[20] Littlejohn MD, Tiplady K, Fink TA, Lehnert K, Lopdell T, Johnson T, et al. Sequence-based association analysis reveals an MGST1 eQTL with pleiotropic effects on bovine milk composition. Sci Rep. 2016;6:25376. 27146958 10.1038/srep25376 PMC4857175

[21] Lopdell TJ, Tiplady K, Struchalin M, Johnson TJJ, Keehan M, Sherlock R, et al. DNA and RNA-sequence based GWAS highlights membrane-transport genes as key modulators of milk lactose content. BMC Genomics. 2017;18:968. 29246110 10.1186/s12864-017-4320-3 PMC5731188

[22] Bolger AM, Lohse M, Usadel B. Trimmomatic: a flexible trimmer for Illumina sequence data. Bioinformatics. 2014;30:2114–20. 24695404 10.1093/bioinformatics/btu170 PMC4103590

[23] Dobin A, Davis CA, Schlesinger F, Drenkow J, Zaleski C, Jha S, et al. STAR: ultrafast universal RNA-seq aligner. Bioinformatics. 2013;29:15–21. 23104886 10.1093/bioinformatics/bts635 PMC3530905

[24] Liao Y, Smyth GK, Shi W. featureCounts: an efficient general purpose program for assigning sequence reads to genomic features. Bioinformatics. 2014;30:923–30. 24227677 10.1093/bioinformatics/btt656

[25] Love MI, Huber W, Anders S. Moderated estimation of fold change and dispersion for RNA-seq data with DESeq2. Genome Biol. 2014;15:550. 25516281 10.1186/s13059-014-0550-8 PMC4302049

[26] Fink T, Lopdell TJ, Tiplady K, Handley R, Johnson TJJ, Spelman RJ, et al. A new mechanism for a familiar mutation - bovine DGAT1 K232A modulates gene expression through multi-junction exon splice enhancement. BMC Genomics. 2020;21:591. 32847516 10.1186/s12864-020-07004-z PMC7449055

[27] Lopdell TJ, Hawkins V, Couldrey C, Tiplady K, Davis SR, Harris BL, et al. Widespread cis-regulation of RNA editing in a large mammal. RNA. 2019;25:319–35. 30530731 10.1261/rna.066902.118 PMC6380278

[28] Prowse-Wilkins CP, Lopdell TJ, Xiang R, Vander Jagt CJ, Littlejohn MD, Chamberlain AJ, et al. Genetic variation in histone modifications and gene expression identifies regulatory variants in the mammary gland of cattle. BMC Genomics. 2022;23:815. 36482302 10.1186/s12864-022-09002-9 PMC9733386

[29] Li H, Durbin R. Fast and accurate short read alignment with Burrows-Wheeler transform. Bioinformatics. 2009;25:1754–60. 19451168 10.1093/bioinformatics/btp324 PMC2705234

[30] Zhang Y, Liu T, Meyer CA, Eeckhoute J, Johnson DS, Bernstein BE, et al. Model-based analysis of ChIP-seq (MACS). Genome Biol. 2008;9:R137. 18798982 10.1186/gb-2008-9-9-r137 PMC2592715

[31] Lopdell TJ, Trevarton AJ, Moody J, Prowse-Wilkins CP, Knowles S, Tiplady K, et al. A common regulatory haplotype doubles lactoferrin concentration in milk. Genet Sel Evol. 2024;56:22. 38549172 10.1186/s12711-024-00890-x PMC11234695

[32] McLaren W, Gil L, Hunt SE, Riat HS, Ritchie GR, Thormann A, et al. The Ensembl variant effect predictor. Genome Biol. 2016;17:122. 27268795 10.1186/s13059-016-0974-4 PMC4893825

[33] Karczewski KJ, Francioli LC, Tiao G, Cummings BB, Alfoldi J, Wang Q, et al. The mutational constraint spectrum quantified from variation in 141,456 humans. Nature. 2020;581:434–43. 32461654 10.1038/s41586-020-2308-7 PMC7334197

[34] VanRaden PM, Tooker ME, O’Connell JR, Cole JB, Bickhart DM. Selecting sequence variants to improve genomic predictions for dairy cattle. Genet Sel Evol. 2017;49:32. 28270096 10.1186/s12711-017-0307-4 PMC5339980

[35] Moghaddar N, Khansefid M, van der Werf JHJ, Bolormaa S, Duijvesteijn N, Clark SA, et al. Genomic prediction based on selected variants from imputed whole-genome sequence data in Australian sheep populations. Genet Sel Evol. 2019;51:72. 31805849 10.1186/s12711-019-0514-2 PMC6896509

[36] Cheruiyot EK, Haile-Mariam M, Cocks BG, Pryce JE. Improving genomic selection for heat tolerance in dairy cattle: current opportunities and future directions. Front Genet. 2022;13: 894067. 35769985 10.3389/fgene.2022.894067 PMC9234448

[37] Jeong S, Kim JY, Kim N. GMSTool: GWAS-based marker selection tool for genomic prediction from genomic data. Sci Rep. 2020;10:19653. 33184432 10.1038/s41598-020-76759-y PMC7665227

[38] Heinrich F, Lange TM, Kircher M, Ramzan F, Schmitt AO, Gultas M. Exploring the potential of incremental feature selection to improve genomic prediction accuracy. Genet Sel Evol. 2023;55:78. 37946104 10.1186/s12711-023-00853-8 PMC10634161

[39] Xiang R, Hayes BJ, Vander Jagt CJ, MacLeod IM, Khansefid M, Bowman PJ, et al. Genome variants associated with RNA splicing variations in bovine are extensively shared between tissues. BMC Genomics. 2018;19:521. 29973141 10.1186/s12864-018-4902-8 PMC6032541

[40] Guo Z, Magwire MM, Basten CJ, Xu Z, Wang D. Evaluation of the utility of gene expression and metabolic information for genomic prediction in maize. Theor Appl Genet. 2016;129:2413–27. 27586153 10.1007/s00122-016-2780-5

[41] Koufariotis LT, Chen YP, Stothard P, Hayes BJ. Variance explained by whole genome sequence variants in coding and regulatory genome annotations for six dairy traits. BMC Genomics. 2018;19:237. 29618315 10.1186/s12864-018-4617-x PMC5885354

[42] Christensen OF, Börner V, Varona L, Legarra A. Genetic evaluation including intermediate omics features. Genetics. 2021;219:iyab130. 34849886 10.1093/genetics/iyab130 PMC8633135

[43] Zhao T, Zeng J, Cheng H. Extend mixed models to multilayer neural networks for genomic prediction including intermediate omics data. Genetics. 2022;221:iyac034. 35212766 10.1093/genetics/iyac034 PMC9071534

